# *In vitro* antioxidant and anti-inflammatory activities and total polyphenol and flavonoid contents of *Anadenanthera colubrina* from northern Peru

**DOI:** 10.17843/rpmesp.2026.431.15207

**Published:** 2026-03-27

**Authors:** Edert Abel Samame-Caramutti, Luis Salcedo-Valdez, Silvia Suárez-Cunza

**Affiliations:** 1 Universidad Nacional Mayor de San Marcos, Facultad de Medicina, Instituto de Investigación de Bioquímica y Nutrición.

**Keywords:** Anti-Inflammatory Agents, Antioxidants, Flavonoids, In Vitro Techniques, Polyphenols

## Abstract

**Objectives.:**

To evaluate the in vitro antioxidant and anti-inflammatory activity of the hydroethanolic extract of the bark of *Anadenanthera colubrina* from northern Peru, as well as its total polyphenol (TPC) and flavonoid (TFC) contents.

**Materials and methods.:**

Antioxidant activity was evaluated using chemical assays for 2,2-diphenyl-1-picrylhydrazyl (DPPH^●^) radical scavenging and ferric reducing antioxidant power (FRAP), as well as by measuring lipid peroxidation (formation of thiobarbituric acid reactive substances, TBARS) and H2O2-induced hemolysis. Anti-inflammatory activity was determined through heat-induced ovalbumin denaturation and hypotonicity-induced hemolysis assays.

**Results.:**

The extract showed high antioxidant activity in both the DPPH^●^ radical scavenging assay (747.6 mg Trolox equivalents/gram of dry extract) and the FRAP assay (435.9 mg FeSO_4_ equivalents/gram of dry extract). The extract (50 to 1000 µg/mL) inhibited lipid peroxidation, H2O2-induced hemolysis, and ovalbumin denaturation. No activity was observed in the hypotonicity-induced hemolysis assay. TPC and TFC were 642.7 mg gallic acid equivalents/gram and 416.2 mg catechin equivalents/gram of dry extract, respectively.

**Conclusions.:**

These activities were reported for the first time for *A. colubrina* from northern Peru, suggesting that the hydroethanolic extract of *A. colubrina* bark possesses significant preliminary in vitro antioxidant and anti-inflammatory properties and that the high polyphenol and flavonoid contents may be responsible, at least partially, for these activities.

## INTRODUCTION

*Anadenanthera colubrina* (Vell.) Brenan is a South American Fabaceae widely used in folk medicine due to its known anti-inflammatory properties ^1^, which is why it is considered a potential source of compounds with biological activity. One of the most used parts is the bark, mainly in the form of syrup, decoction, infusion, and maceration. These preparations are used to treat a variety of conditions, ranging from common problems such as allergies and inflammation to serious diseases such as anemia, respiratory diseases (for example, bronchitis and tuberculosis), and cancer; they also serve as healing agents ^2^. In this sense, several studies have reported the antioxidant properties of the bark ^3^^,^^4^, which may explain its anti-inflammatory activity in vivo ^5^ and in vitro ^6^. *A. colubrina* grows in the tropical rainforests and seasonally dry forests of Peru between 600 and 2000 m above sea level ^7^. Although this species has been widely used in South American folk medicine ^8^, it has not received due importance in northern Peru, where it is primarily used for wood production ^7^. Considering that there is scarce scientific information supporting its traditional use ^9^ and that the native variety from Peru has not been previously studied, the objective of this study was to determine the in vitro antioxidant and anti-inflammatory activities of the bark of *A. colubrina* from northern Peru, as well as its total polyphenol and flavonoid content

**KEY MESSAGES**

Motivation for the study. Despite its widespread use in South American folk medicine, *Anadenanthera colubrina* from northern Peru is primarily used as timber, largely ignoring its medicinal potential.

Main findings. The hydroethanolic extract of *A. colubrina* bark presented substantial preliminary in vitro antioxidant and anti-inflammatory activities, attributable, at least partially, to its high polyphenol and flavonoid contents.

Public health implications. These findings demonstrate that *A. colubrina* from northern Peru has significant potential for preventing and treating inflammatory conditions and will benefit local inhabitants, who are often unaware of the medicinal properties of our natural resources, thereby reinforcing its value both in Peru and abroad.

## MATERIALS AND METHODS

### Reagents and standards

2,2-diphenyl-1-picrylhydrazyl (DPPH^●^), Folin-Ciocalteu phenol reagent (2N), gallic acid, (+)-catechin, (+)-6-hydroxy-2,5,7,8-tetramethylchromane-2-carboxylic acid (Trolox), 2-thiobarbituric acid, and sodium monobasic phosphate (> 99%) were obtained from Sigma-Aldrich (MO, USA). Sodium carbonate, sodium chloride (> 99.5%), D(+)-glucose, Perhydrol® 30% H2O2, and anhydrous disodium phosphate (99%) were purchased from Merck (Germany). Citric acid-1-hydrate was obtained from Riedel-de Haën (Germany). Diclofenac sodium was a generous donation from a national pharmaceutical laboratory. All other chemicals used were of analytical grade. All absorbance values were determined using a Thermo Fisher Scientific Genesys 50S UV-Vis spectrophotometer (Waltham, MA, USA) in 1 cm quartz cuvettes.

### Plant material and extraction

The bark of *Anadenanthera colubrina* (3 kg) was collected locally in the primary forest of Las Juntas (Department of Cajamarca, Peru) (5°20’30” S, 78°46’00” W; average altitude 625 m) (10), with the authorization of the local inhabitants. The plant was authenticated by Prof. Eric Rodríguez-Rodríguez, and two reference specimens were deposited in the Herbarium Truxillense of the Universidad Nacional de Trujillo (Trujillo, Peru) (specimen code numbers: 57669-HUT and 57670-HUT) (supplementary material: figure 1).

The hydroethanolic extract was obtained using methods reported in the literature for other Fabaceae ^11^^,^^12^, with modifications; briefly, the bark was dried for 14 days at room temperature and ground. A sample of powdered bark (1.4 kg) was extracted through successive exhaustive extraction with 96% ethanol (1:2 w/v) for 48 hours at room temperature under stirring. The crude extracts were combined, filtered, and concentrated to dryness, yielding a reddish-brown dry product (*A. colubrina* bark hydroethanolic extract, EEAC). The extraction yield was 20.7%. The EEAC was stored at 4°C.

The EEAC was completely dissolved in a mixture of 96% ethanol: bidistilled water (1:29), and this solution was used for all assays.

### Physicochemical analysis

The soluble solids content of the EEAC (2 mg/mL) was determined gravimetrically using an Ohaus® PioneerTM analytical balance (120 g, d = 1 mg; Parsippany, NJ, USA), and by measuring the refractive index (solvent: 96% ethanol: bidistilled water, 1:29) using an ATAGO® PAL-α handheld refractometer (Tokyo, Japan). The pH was obtained using pH indicator strips (Merck, Germany).

### Qualitative phytochemical analysis and total polyphenol (TPC) and flavonoid (TFC) content:

Qualitative phytochemical analysis was performed according to Lock de Ugaz ^13^. TPC was determined by the Folin-Ciocalteu method ^14^. The EEAC (40-120 µg/mL, 0.1 mL) was incubated with 10% Folin reagent (0.5 mL) and 7.5% w/v Na2CO3 (0.4 mL) at room temperature for 30 minutes. Absorbance changes were read at 765 nm. Results were expressed in mg of gallic acid equivalents (GAE)/g of dry EEAC, and were calculated from a calibration curve using gallic acid as a standard (0-100 μg/mL) and the formula: 

TPC: mg GAE / g EEAC = (A_s * DF) / (m * c)

where *As* is the absorbance with the sample (EEAC), *m* is the slope of the gallic acid standard curve equation, *c* is the concentration of the sample (EEAC), and DF is the dilution factor.

TFC was estimated as described by Zhishen *et al*. ^15^. The EEAC (50-250 µg/mL, 0.5 mL) was incubated, successively, with 5% NaNO2 (w/v; 0.15 mL) for 5 minutes, 2.5% AlCl3 (w/v; 0.25 mL) for 6 minutes, and 1M NaOH (0.25 mL) for 10 minutes. Changes in absorbance were read at 510 nm. Results were expressed as mg of catechin equivalents (CE)/g of dry EEAC, and were calculated from a calibration curve using catechin as a standard (0-72 µg/mL) and the formula:

TFC: mg CE / g EEAC = (*A_s* * DF) / (m * c)

where *m* is the slope of the catechin standard curve equation, and *As*, *c*, and DF are as indicated before.

### Antioxidant activity (Chemical and biological assays):

DPPH^●^ free radical scavenging assay

The DPPH^●^ free radical scavenging activity was evaluated according to Brand-Williams *et al*. ^16^. Briefly, a DPPH^●^ solution (20 mg/dL) prepared in ethanol was stirred for 40 minutes; this stock solution was kept in the dark at 4°C. The initial absorbance of the working solution was adjusted to 0.9 + 0.02 at 517 nm (DPPH^●^ concentration: 78.5 μM) with the same solvent, and was used to prepare the control (absorbance: 0.6 + 0.02). The EEAC (5-20 µg/mL, 0.4 mL) was incubated with the DPPH^●^ working solution (0.8 mL) for 30 minutes in the dark. Changes in absorbance were read at 517 nm. The radical scavenging percentage was calculated as: 

% radical scavenging = ((A_c - A_s) * 100) / A_c

where A*c* and A*s* are the absorbances without (negative control) and with the sample, respectively.

The inhibitory concentration values of EEAC required to scavenge 50% of the initial free radical (IC50) were calculated by linear regression. The Trolox equivalent antioxidant capacity (TEAC- DPPH^●^) was expressed as mg TE/g of dry EEAC, and was calculated from a calibration curve using Trolox as a standard (0-15 µg/mL) and the formula: 

TEAC- DPPH^●^: mg TE / g EEAC = IC50 Trolox / IC50 EEAC

Ferric reducing antioxidant power (FRAP) assay

The assay was performed according to Benzie and Strain ^17^. The EEAC (40-160 μg/mL, 50 μL) was added to 950 μL of the FRAP working solution (10mM 2,4,6-tris(2-pyridyl)-s-triazine (TPTZ) in 40mM HCl, 20mM FeCl3 in bidistilled water, and 0.3M acetate buffer, pH 3.6, in a ratio of 1:1:98). The reaction mixtures were incubated for 10 min. Changes in absorbance were read at 593 nm. Results were expressed as mg FeSO4 equivalents/g of dry EEAC, and were calculated from a calibration curve using FeSO4 as a standard (0-91 µg/mL) and the formula: 

FRAP: mg Eq FeSO_4 / g EEAC = (A_*s* * DF) / (*m* * c)

where A*s* is the absorbance with the sample (EEAC), *m* is the slope of the FeSO4 standard curve equation, c is the concentration of the sample (EEAC), and DF is the dilution factor.

Lipid peroxidation inhibition assay using thiobarbituric acid reactive substances (TBARS)

Oxidative stress, expressed as TBARS, was measured by the method of Buege and Aust ^18^, with modifications. Frozen liver tissue from a Holtzman rat (previously perfused with ice-cold 0.154M KCl) was homogenized in cold phosphate buffered saline (PBS, pH 7.4). Lipid peroxidation was initiated by adding 4 mM ascorbic acid (0.045 mL) and 2 mM FeSO4 (0.015 mL) successively to the 5% liver homogenate (w/v, 0.9 mL). Subsequently, different concentrations of EEAC (100-1000 μg/mL, 0.06 mL) or PBS (pH 7.4, 0.06 mL and 0.12 mL for the induced stress and control groups, respectively) were included in the reaction mixtures; then, these were incubated for 30 minutes at room temperature. Afterward, each reaction mixture (0.3 mL) was mixed with 20% trichloroacetic acid (0.6 mL), heated in boiling water for 15 minutes, cooled with tap water, mixed with 0.67% thiobarbituric acid (TBA in 0.25 N HCl, 0.9 mL) and heated again in boiling water for 30 minutes. After cooling with cold tap water, each tube was centrifuged (5488xg for 10 minutes), the supernatant was collected, and the absorbance was determined at 535 nm. 

Antioxidant activity was expressed as a percentage of lipid peroxidation inhibition and as TBARS concentration. The percentage of lipid peroxidation inhibition was calculated as follows:

% lipid peroxidation inhibition = ((A_*c* - A_*s*) * 100) / A_c

where Ac is the absorbance of the induced stress group (0% lipid peroxidation inhibition, negative control) and As is the absorbance with the sample (EEAC). 

The TBARS concentration was expressed as nmol of TBARS/mg of liver tissue and was calculated as follows: 

TBARS: (nmol TBARS/mg liver tissue) = (*A_s* * V_t) /

(ε * l * Vs * CH)

where *As* is the absorbance of each reaction mixture, Vt is the final volume of the reaction mixture, *ε* is the molar absorption coefficient of the MDA-TBA2 complex at 535 nm (1.56 x 105 M-1 cm-1), l is the optical path of the cuvette (1 cm), Vs is the sample volume in the reaction mixture, and CH is the rat liver homogenate concentration.

H_2_O_2_-induced oxidative hemolysis assay

The assay was performed following the method of Xu *et al*. ^19^, with modifications. Fresh human venous blood (3 mL) was employed from a healthy donor and diluted with sterilized Alsever's solution (2% dextrose, 0.8% sodium citrate, 0.05% citric acid, and 0.42% NaCl in bidistilled water). The solution was centrifuged (625xg for 3 minutes) and washed three times with the same volume of vehicle (cold 1X PBS, pH 7.4) to separate human red blood cells (RBCs) from the white blood cell layer, plasma, and free hemoglobin (Hb) released from injured RBCs. A 5% RBC suspension (in ice-cold Alsever’s solution, 0.1 mL) was mixed with EEAC (50-150 μg/mL, 0.45 mL) or vehicle (0.45 mL and 0.9 mL for the induced stress and control groups, respectively). The reaction mixtures were preincubated at 37 °C for 15 minutes. Then, 166mM H2O2 (in vehicle, 0.45 mL) was added, the reaction mixture was incubated at 37°C for 4 hours and centrifuged at 625xg for 10 minutes. The Hb content in the supernatant was estimated at 405 nm. After each assay, the RBC solution was stored at 4°C for a maximum of 72 h to ensure viability and the level of cellular antioxidants. The percentage of hemolysis prevention was calculated as follows: 

% hemolysis prevention = ((*A_c - A_s*) * 100) / *A_c*

where A*c* is the absorbance of the induced stress group (0% hemolysis prevention, negative control) and A*s* is the absorbance with the sample (EEAC).

### Anti-inflammatory activity

Inhibition of ovalbumin denaturation

The assay was carried out as described by Mizushima and Kobayashi ^20^ and Chandra ^et al^. ^21^, with modifications. The reaction mixture (1.3 mL) consisted of fresh ovalbumin: PBS (pH 6.4) (1:1, 0.1 mL), PBS (pH 6.4, 0.7 mL), and different concentrations of the EEAC (125-750 μg/mL, 0.5 mL). Bidistilled water and diclofenac sodium (0.5-3 mg/mL) were used for the induced stress group and as a standard, respectively. The reaction mixtures were incubated at 37 + 1°C for 20 minutes and subsequently heated at 65 + 2°C for 5 minutes to induce denaturation. After cooling, absorbance changes were read at 660 nm. The percentage of protein denaturation inhibition was calculated as:

% protein denaturation inhibition = ((A_c - A_s) * 100) / A_c

where A*c* is the absorbance of the induced stress group (0% protein denaturation inhibition, negative control) and A*s* is the absorbance with the sample.

Human red blood cell (RBC) membrane stabilization

The assay was performed according to the procedures described by Lavanya *et al.*^22^ and Torres Carro *et al*. ^23^, with modifications. Fresh human venous blood (3 mL) from a healthy donor was used and diluted with an equal volume of sterilized Alsever's solution. The solution was centrifuged (5,488xg for 10 minutes) and the cell pack was washed five times with an equal volume of isotonic saline solution (0.85% NaCl, pH 7.2) to prepare a 10% (v/v) RBC suspension with isotonic saline solution. The suspension was stored at 4°C. The reaction mixture (1.5 mL) consisted of the 10% RBC suspension (0.2 mL), 0.14M sodium phosphate buffer (pH 7.4, 0.333 mL), and different concentrations of the EEAC (250-750 μg/mL, 0.267 mL). Bidistilled water (0.7 mL) was used to induce hypotonic hemolysis. Vehicle (96% ethanol: bidistilled water, 1:29) and diclofenac sodium (1-3 mg/mL) were used for the induced stress group and as a standard, respectively. The reaction mixtures were incubated at 37 + 1°C for 30 minutes and then centrifuged at 650xg for 3 minutes. The Hb content in the supernatant was measured at 550 nm. After each test, the RBC solution was stored at 4°C for a maximum of 72 h to ensure cell viability. The percentage of hemolysis prevention was calculated as follows: 

% hemolysis prevention = ((*A_c - A_s*) * 100) / A*_c*

where A*c* is the absorbance of the induced stress group (0% hemolysis prevention, negative control) and As is the absorbance with the sample.

Statistical analysis

The assays were performed in three independent experiments, each conducted in duplicate or triplicate. All analyses were performed using Minitab Statistical Software and GraphPad Prism (version 8.0.1). Normality and homogeneity of data variances were evaluated using Shapiro-Wilk and Levene tests, respectively (p>0.05 in both). Since the data were normally distributed, a one-way ANOVA followed by Tukey's post-hoc test was used. Results are expressed as mean + standard deviation, and statistical significance was defined as p<0.05.

Ethical aspects

The study was carried out in accordance with the Declaration of Helsinki ^24^ and established ethical standards. A human blood sample was obtained from a healthy volunteer at the Institute of Biochemistry and Nutrition Research (IIBN), UNMSM, after receiving written informed consent. Regarding the animal component, we used liver tissue from a rat without prior treatment as a secondary use of samples from a parallel IIBN project, following the 3Rs principles (Replacement, Reduction, and Refinement) to avoid the sacrifice of additional animals. This project was approved by the Ethics Committee of the Faculty of Medicine of the UNMSM (Ethics Approval No. 0185-2024), in accordance with the Ethical Evaluation Act of Research Studies.

## RESULTS

### Physicochemical analysis

The soluble solids content was 1.606 + 0.3 mg/mL and 1.711 + 0.2 mg/mL, according to the refractive index and gravimetric analysis, respectively. The pH was 5.5-6.0 (slightly acidic).

### Qualitative phytochemical analysis, TPC and TFC

As presented in supplementary material: table1, the EEAC has a high content of phenolic compounds (tannins and flavonoids), low content of glycosides, anthraquinones, and anthrones, and undetectable or null presence of alkaloids, triterpenes, steroids, and saponins. 

The determination of total polyphenols and flavonoids confirmed their abundance, with 642.7 + 17 mg (3.78 + 0.1 mmol) GAE/g and 416.2 + 20 mg (1.6 + 0.08 mmol) CE/g dry extract, respectively; that is, TPC represents approximately 65% in GAE of the extract, which mainly includes flavonoids (around 41% in CE of the extract). The flavonoid/polyphenol ratio was 0.65.

### Antioxidant activity (Chemical and biological assays)

DPPH^●^ free radical scavenging assay

**Figure 1:**
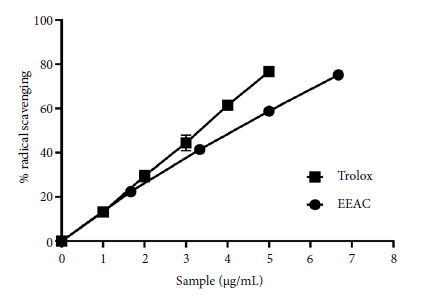
Free radical scavenging effects of DPPH ^●^ (78.5 μM) of the hydroethanolic extract of *Anadenanthera colubrina* bark (EEAC) and Trolox. Results are presented as mean + standard deviation (n = 3), representative of two replicates

The EEAC showed an IC50 value (4.37 µg/mL) close to the Trolox standard (3.26 µg/mL, Figure 1 and supplementary material: table 2). The aforementioned value represents a TEAC- DPPH^●^ of 747.55 mg (2.99 mmol) Trolox equivalents/g dry EEAC (supplementary material: table 2).

FRAP assay

**Figure 2:**
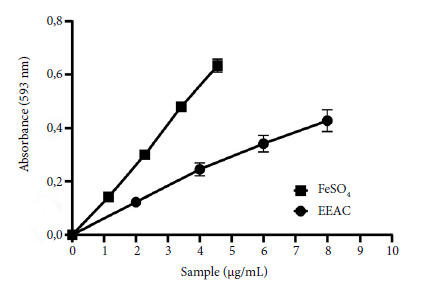
Reactivity of the hydroethanolic extract of *Anadenanthera colubrina* bark (EEAC) and ferrous sulphate in the ferric reducing antioxidant power (FRAP) assay. Results are presented as mean + standard deviation (n = 3), representative of two replicates.

The EEAC exhibited an antioxidant capacity of 435.88 mg (2.87 mmol) FeSO4 equivalents/g dry EEAC (Figure 2 and supplementary material: table 2).

Lipid peroxidation inhibition assay using TBARS

**Figure 3:**
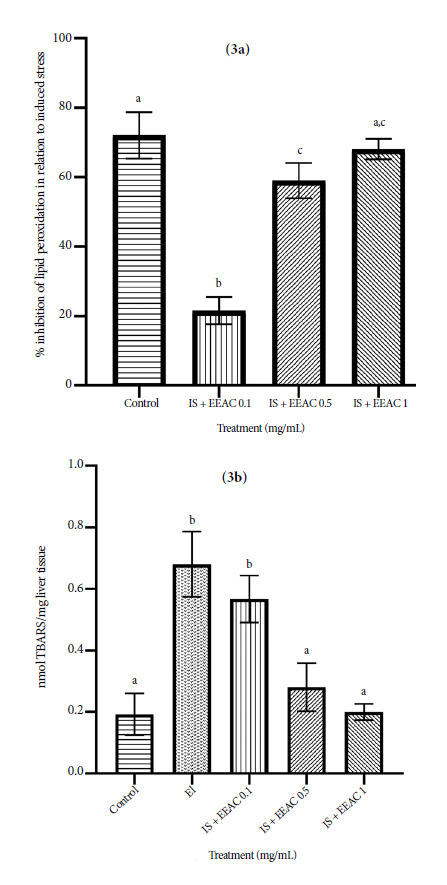
(a) Percentage of inhibition of lipid peroxidation by the hydroethanolic extract of *Anadenanthera colubrina* bark (EEAC) and (b) effect of EEAC on TBARS formation in rat liver homogenate. Results are presented as mean + standard deviation (n = 3), representative of two replicates, and were evaluated using one-way analysis of variance and Tukey's test (p<0.05). Statistical differences are indicated by different letters. IS: induced stress.

All tested concentrations (100, 500, and 1000 μg/mL) were effective in reducing lipid peroxidation in a dose-dependent manner, by 22, 59, and 68%, respectively, with respect to the induced stress (IS) group (Figure 3a). No differences were found between treatment with EEAC at 1000 μg/mL and the Control group (72%).

When expressed as nmol/mg of liver tissue, TBARS production was higher in the IS group than in the EEAC treatments at 500 and 1000 μg/mL, indicating a dependency on the extract concentration. No differences were found between the IS group and EEAC at 100 μg/mL, nor between the Control and EEAC treatments at 500 and 1000 μg/mL (Figure 3b).

H_2_O_2_-induced oxidative hemolysis assay

**Figure 4:**
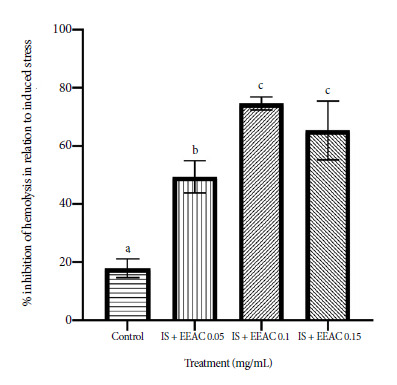
Inhibition of oxidative hemolysis by H2O2 by the hydroethanolic extract of *Anadenanthera colubrina* bark (EEAC). Results are presented as mean + standard deviation (n = 3), representative of three replicates, and were evaluated using one-way analysis of variance and Tukey's test (p<0.05). Statistical differences are indicated by different letters. IS: induced stress.

The EEAC, administered at 50, 100, and 150 μg/mL, decreased hemolysis caused by H2O2 when compared with the IS group (Figure 4). Hemolysis prevention was dose-dependent only for the 50 and 100 μg/mL treatments (49 and 75% inhibition, respectively) since the 150 μg/mL treatment caused an inhibition (65%) similar to that observed for the 100 μg/mL group. Interestingly, the percentage of inhibition for the three concentrations of EEAC was much higher than that reported for the Control group (18%).

### Anti-inflammatory activity

Inhibition of ovalbumin denaturation

**Figure 5:**
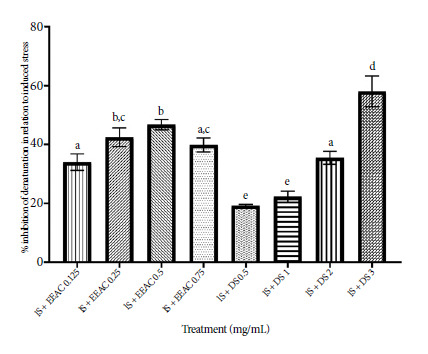
Inhibition of ovalbumin denaturation by the hydroethanolic extract of *Anadenanthera colubrina* bark (EEAC). Diclofenac sodium (DS) was used as standard. Results are presented as mean + standard deviation (n = 3), representative of three replicates, and were evaluated using one-way analysis of variance and Tukey's test (p<0.05). Statistical differences are indicated by different letters. IS: induced stress.

A dose-dependent inhibition of ovalbumin denaturation was observed with EEAC at 125, 250, and 500 μg/mL, at 34, 42, and 47%, respectively, with respect to the IS group (Figure 5). Curiously, the extract at 750 μg/mL reduced denaturation by only 40%, lower than the inhibition observed at 500 μg/mL. In any case, the EEAC at all analyzed concentrations showed higher inhibition than the non-steroidal anti-inflammatory drug (NSAID) diclofenac sodium at 0.5 and 1 mg/mL (19 and 22%, respectively), but lower than said reference at 3 mg/mL (58%). The EEAC at 250 and 500 μg/mL showed better inhibition of protein denaturation than diclofenac sodium at 2 mg/mL.

RBC membrane stabilization

EEAC at 250, 500, and 750 μg/mL did not have a significant effect on hypotonicity-induced hemolysis. In this aspect, the extract at 250 and 500 μg/mL produced slightly higher hemolysis than the IS group (6 and 5%, respectively). Likewise, the concentration of 750 μg/mL inhibited hemolysis by only 6%. Despite this, none of the concentrations showed a significant difference compared to the IS group. On the other hand, diclofenac sodium inhibited hemolysis between 29% and 42% at the concentrations used (1, 2, and 3 mg/mL).

## DISCUSSION

The results concerning the phytochemical content of the EEAC are consistent with previous reports on the bark of *A. colubrina*, i.e., the hydroalcoholic extract is rich in phenolic compounds ^4^^,^^25^, which include both tannins and flavonoids ^6^. However, some previous studies are inconsistent with the results of this phytochemical analysis. Silva *et al*. ^26^ reported the presence of alkaloids and saponins, as well as the absence of flavonoids, anthraquinones, and glycosides in the hydroalcoholic bark extract. On the other hand, Sá *et al*. ^27^ revealed that the hydroalcoholic bark extract contains phenolic compounds, tannins, and sugars, but flavonoids, steroids, or terpenoids were not detected. Additionally, Pessoa *et al*. ^28^ reported the presence of reducing sugars, saponins, triterpenes, and steroids. These differences would indicate that edaphic and environmental conditions, as well as differences in extraction procedures, affect the biosynthesis or the presence of secondary metabolites despite being the same species.

Interestingly, the hydroethanolic bark extract of the Brazilian variety reported by Mota *et al*. ^4^^)^ showed higher TPC and TFC values than those of the native *A. colubrina* variety from northern Peru investigated in this study (682 mg GAE/g extract and 445 mg CE/g extract for the Brazilian variety vs. 643 mg GAE/g extract and 416 mg CE/g extract for the Peruvian variety, respectively). Despite this, the Peruvian variety has higher antioxidant properties than the aforementioned Brazilian variety.

The EEAC showed good behavior in two antioxidant assays with different mechanisms of action, single electron transfer for the FRAP assay and hydrogen atom transfer or single electron transfer for the DPPH^●^ radical scavenging assay ^29^. Moreover, as reported by Desmarchelier *et al*. ^30^, the EEAC decreased the formation of malondialdehyde and other TBARS as by-products of lipid peroxidation in rat liver homogenates and, furthermore, reduced the oxidative damage caused by H_2_O_2_ in an RBC model, showing the ability of the EEAC to decrease oxidative stress *in vitro*.

These results suggest that the Peruvian variety of *A. colubrina* possesses the antioxidant activity previously reported for the bark. This antioxidant activity is likely due to the phytochemical content of the EEAC, mainly phenolic compounds (tannins and flavonoids) ^4^. Nonetheless, there are some contradictory results regarding polyphenol production by *A. colubrina* under different environmental conditions ^31^, which in turn could explain some discrepancies between the present study and previous reports ^4^^,^^26^^,^^32^.

It is noteworthy that the Peruvian variety exhibited a higher TEAC- DPPH^●^ value and a lower IC_50_ than the Brazilian variety reported by the aforementioned study by Mota *et al*. ^4^ (269 mg Trolox equivalents/g extract and 13 μg/mL for the Brazilian variety vs. 748 mg Trolox equivalents/g extract and 4 μg/mL for the Peruvian variety, respectively). Consequently, the antioxidant properties (TEAC- DPPH^●^ and IC_50_) of the Brazilian variety would be considered only moderately intense ^4^, and lower than those of the Peruvian variety reported here.

These findings indicate that, as previously mentioned, in addition to differences in extraction techniques, edaphic and environmental conditions influence the presence of secondary metabolites and, consequently, the biological activity of the extracts. This is particularly evident when comparing these results with the studies by Mota *et al*. (2017) ^4^ and Silva *et al*. (2020) ^26^ mentioned above, since all these studies used hydroethanolic extracts. Worth noting is the report by Silva *et al*. (2020) ^26^, which revealed the presence of compounds absent in the EEAC, as well as the absence of flavonoids, which were abundant here. Furthermore, their extract exhibited an IC50 of 20 μg/mL in the DPPH assay, much higher than the 4.37 μg/mL reported in the present work.

The different extraction methods also contribute to variations in phytochemical content and antioxidant activity. In this context, several studies have reported the activity of hydromethanolic extracts of *A. colubrina* bark; for example, Sá *et al.* (2016) reported reduced extraction of flavonoids ^27^, which were abundant in the present study. Additionally, Pessoa et al. (2012) detected molecules such as saponins, triterpenes, and steroids ^28^ that were absent in this investigation. This type of extract also showed higher antioxidant activity in the TBARS assay (IC_50_ of 62 μg/mL) ^30^ compared to the present study (IC_50_> 100 μg/mL), but lower activity in the DPPH assay (IC50 of 73 μg/mL) ^32^.

The use of aqueous extracts of *A. colubrina* bark also yielded interesting results. Specifically, Damascena *et al*. (2014) ^3^ revealed an IC_50_ of 8.63 μg/mL in the DPPH assay, indicating lower antioxidant activity compared to this work. Conversely, aqueous extracts showed variable antioxidant activities in the TBARS assay, which were similar ^3^ or higher ^30^ than those observed with the EEAC.

The ovalbumin denaturation assay was used to evaluate the anti-inflammatory activity of *A. colubrina*. This method is based on the stabilizing action of NSAIDs on heat-induced protein coagulation ^20^. Since the denaturation of tissue proteins is capable of initiating immune responses and causing inflammatory disorders, it has been suggested that compounds that prevent protein denaturation could have anti-inflammatory potential ^33^. In this aspect, the EEAC prevented heat-induced ovalbumin denaturation to a greater extent than the NSAID diclofenac sodium, which has a known protective activity against protein denaturation.

To corroborate this anti-inflammatory effect, the RBC membrane stabilization assay was performed. This assay is based on the similarity between erythrocyte and lysosomal membranes. Lysosomal membrane stabilization is desirable, since lysosomes can release their hydrolytic enzymes into affected tissues during the inflammatory response. The effect of any extract on erythrocyte stabilization could be extrapolated to lysosomal membrane stabilization and, therefore, the anti-inflammatory activity of an extract can be evaluated by preventing hemolysis ^34^.

No activity was observed at the same concentrations used in the denaturation assay; on the contrary, the EEAC increased hypotonicity-induced hemolysis, although these results were not entirely unexpected. The EEAC is rich in tannins (supplementary material: table 1), which could have hemolytic activity on RBCs ^35^. In this sense, Rocha *et al*. ^25^ reported that the hydroalcoholic extract of *A. colubrina* bark was slightly toxic to RBCs, even at low concentrations (0.25 to 32 mg/mL). From this perspective, the high tannin content of the EEAC could explain the hemolysis observed in the present study. As described by Deng *et al*. (2019) ^35^, high concentrations of some tannins could cause changes in erythrocyte morphology and hemolysis, possibly due to the phenolic hydroxyl groups of tannins, which adsorb onto the cell membrane, causing deformations that lead to its rupture. It is well known that tannins bind to and alter cell membrane proteins and lipids, thereby modifying their permeability and causing cell damage and lysis, which also affects blood coagulation.

Although these results would indicate that the EEAC is a potential source of anti-inflammatory compounds, especially those related to protein denaturation, it must be considered that the assays were performed in vitro, and do not necessarily show what would occur in vivo, as the bioavailability of active compounds, such as tannins, must be considered. However, it is promising that *A. colubrina* has demonstrated better inhibitory activity than an NSAID like diclofenac sodium, which can inhibit protein denaturation in a dose-dependent manner.

In conclusion, the biological properties of *Anadenanthera colubrina* from northern Peru were reported for the first time. In this sense, the hydroethanolic bark extract (EEAC) possesses strong preliminary in vitro antioxidant activity, as well as a high content of total polyphenols and flavonoids. Furthermore, the EEAC prevented heat-induced ovalbumin denaturation to a greater extent than the NSAID diclofenac sodium. These results suggest that *A. colubrina* from northern Peru is a good source of compounds with biological activity, benefiting the inhabitants of those areas, who might be unaware of the medicinal properties of their own resources. Furthermore, a practical methodology for evaluating the antioxidant and anti-inflammatory activities of Peruvian medicinal plants is provided.

As an additional recommendation for the use of *A. colubrina*, it is suggested that excess saponins and tannins be removed using standardized extraction methods to optimize its biological properties, since these compounds, although present in the bark, can exert toxic effects at high concentrations. Further research is needed to determine the safety and specific mechanisms of action of *A. colubrina*, and to purify and identify the potential molecules responsible for the observed pharmacological activity.

## References

[1] Júnior WS, Ladio AH, Albuquerque UP (2011). Resilience and adaptation in the use of medicinal plants with suspected anti-inflammatory activity in the Brazilian Northeast. J Ethnopharmacol.

[2] Macedo JGF, de Menezes IRA, Ribeiro DA, Santos MO, Mâcedo DG, Macêdo MJF (2018). Analysis of the variability of therapeutic indications of medicinal species in the northeast of Brazil: comparative study. Evid Based Complement Alternat Med.

[3] Damascena NP, Souza MTS, Almeida AF, Cunha RS, Damascena NP, Curvello RL (2014). Antioxidant and orofacial anti-nociceptive activities of the stem bark aqueous extract of Anadenanthera colubrina (Velloso) Brenan (Fabaceae). Nat Prod Res.

[4] Mota GS, Sartori CJ, Miranda I, Quilhó T, Mori FA, Pereira H (2017). Bark anatomy, chemical composition and ethanol-water extract composition of Anadenanthera peregrina and Anadenanthera colubrina. PLoS One.

[5] Pimentel VD, Acha BT, Gomes GF, Cardoso JLMS, Costa CLS, Batista NJC (2024). Anti-inflammatory effect of Anadenanthera colubrina var cebil (Griseb.) Altschul in experimental elastase-induced pulmonary emphysema in rats. J Ethnopharmacol.

[6] Maia CMA, Vasconcelos PGS, Pasetto S, Godwin WC, Silva JPRE, Tavares JF (2024). Anadenanthera colubrina regulated LPS-induced inflammation by suppressing NF- B and p38-MAPK signaling pathways. Sci Rep.

[7] Marcelo-Peña JL, Pennington RT, Reynel C, Zevallos P (2010). Guía ilustrada de la flora leñosa de los bosques estacionalmente secos de Jaén, Perú [Illustrated guide to the woody flora of seasonally dry forests of Jaen, Peru],.

[8] Albuquerque UP, Medeiros PM, Almeida ALS, Monteiro JM, Lins Neto EMF, Melo JG (2007). Medicinal plants of the caatinga (semi-arid) vegetation of NE Brazil A quantitative approach. J Ethnopharmacol.

[9] Weber CR, Soares CML, Lopes ABD, Silva TS, Nascimento MS, Ximenes ECPA (2011). Anadenanthera colubrina: um estudo do potencial terapêutico [Anadenanthera colubrina: a therapeutic potential study]. Rev Bras Farm.

[10] Tropicos.org (1982). http://www.tropicos.org/NamePage.aspx?nameid=13015659&tab=specimens.

[11] Kondeti VK, Badri KR, Maddirala DR, Thur SK, Fatima SS, Kasetti RB (2010). Effect of Pterocarpus santalinus bark, on blood glucose, serum lipids, plasma insulin and hepatic carbohydrate metabolic enzymes in streptozotocin-induced diabetic rats. Food Chem Toxicol.

[12] Olaleye MT, Akinmoladun AC, Crown OO, Ahonsi KE, Adetuyi AO (2013). Homopterocarpin contributes to the restoration of gastric homeostasis by Pterocarpus erinaceus following indomethacin intoxication in rats. Asian Pac J Trop Med.

[13] Lock de Ugaz O (1994). Investigación fitoquímica: Métodos en el estudio de productos naturales.

[14] Singleton VL, Orthofer R, Lamuela-Raventós RM (1999). Analysis of total phenols and other oxidation substrates and antioxidants by means of folin-ciocalteu reagent. Methods Enzymol.

[15] Zhishen J, Mengcheng T, Jianming W (1999). The determination of flavonoid contents in mulberry and their scavenging effects on superoxide radicals. Food Chem.

[16] Brand-Williams W, Cuvelier ME, Berset C (1995). Use of a free radical method to evaluate antioxidant activity. Lebensm Wiss Technol.

[17] Benzie IF, Strain JJ (1996). The ferric reducing ability of plasma (FRAP) as a measure of "antioxidant power" the FRAP assay. Anal Biochem.

[18] Buege JA, Aust SD (1978). Microsomal lipid peroxidation. Methods Enzymol.

[19] Xu X, He J, Liu G, Diao X, Cao Y, Ye Q (2014). Hemolysis assessment and antioxidant activity evaluation modified in an oxidized erythrocyte model. J Agric Food Chem.

[20] Mizushima Y, Kobayashi M (1968). Interaction of anti-inflammatory drugs with serum proteins, especially with some biologically active proteins. J Pharm Pharmacol.

[21] Chandra S, Chatterjee P, Dey P, Bhattacharya S (2012). Evaluation of in vitro anti-inflammatory activity of coffee against the denaturation of protein. Asian Pac J Trop Biomed.

[22] Lavanya R, Maheshwari SU, Harish G, Raj JB, Kamali S, Hemamalani D (2010). Investigation of in-vitro anti-inflammatory, anti-platelet and anti-arthritic activities in the leaves of Anisomeles malabarica Linn. Res J Pharm Biol Chem Sci.

[23] Torres Carro R, D'Almeida RE, Isla MI, Alberto MR (2016). Antioxidant and anti-inflammatory activities of Frankenia triandra (J Rémy) extracts. S Afr J Bot.

[24] Asociación Médica Mundial (2024). Declaración de Helsinki de la AMM: Principios éticos para las investigaciones médicas con participantes humanos..

[25] Rocha EALSS, Medeiros ACD, Castro RD, Rosalen PL, Saraiva KLA, Godoy GP (2017). Antifungal activity, phytochemical characterization and thermal profile of Anadenanthera colubrina (Vell.) Brenan. Pesq Bras Odontoped Clin Integr.

[26] Silva ELGS, Aguiar HTV, Freitas RF (2020). Estudo fitoquímico, atividade antioxidante e tóxica da casca da Anadenanthera colubrina (Vell.) Brenan. Biodiversidade.

[27] Sá JLF, Siqueira WN, Silva HAMF, Santos MLO, Santos FTJ, Silva LRS (2016). Evaluation of molluscicidal activity of Anadenanthera colubrina extracts on adult mollusc and embryos of the species Biomphalaria glabrata (Say, 1818). Sci Plena.

[28] Pessoa WS, Estevão LRM, Simões RS, Barros MEG, Mendonça FS, Baratella-Evêncio L (2012). Effects of angico extract (Anadenanthera colubrina var cebil) in cutaneous wound healing in rats. Acta Cir Bras.

[29] Prior RL, Wu X, Schaich K (2005). Standardized methods for the determination of antioxidant capacity and phenolics in foods and dietary supplements. J Agric Food Chem.

[30] Desmarchelier C, Romão RL, Coussio J, Ciccia G (1999). Antioxidant and free radical scavenging activities in extracts from medicinal trees used in the 'Caatinga' region in northeastern Brazil. J Ethnopharmacol.

[31] Araújo TAS, Almeida VTN, Solon LGS, Silva GA, Almeida MG, Costa JGM (2015). Does rainfall affect the antioxidant capacity and production of phenolic compounds of an important medicinal species. Ind Crops Prod.

[32] Melo JG, Araújo TAS, Almeida VTN, Cabral DLV, Rodrigues MD, Nascimento SC (2010). Antiproliferative activity, antioxidant capacity and tannin content in plants of semi-arid northeastern Brazil. Molecules.

[33] Aidoo DB, Konja D, Henneh IT, Ekor M (2021). Protective effect of Bergapten against human erythrocyte hemolysis and protein denaturation in vitro. Int J Inflam.

[34] Saleem TKM, Azeem AK, Dilip C, Sankar C, Prasanth NV, Duraisami R (2011). Anti-inflammatory activity of the leaf extracts of Gendarussa vulgaris Nees. Asian Pac J Trop Biomed.

[35] Deng L, Qi Y, Liu Z, Xi Y, Xue W (2019). Effect of tannic acid on blood components and functions. Colloids Surf B Biointerfaces.

