# Supplementary material for: In vitro antioxidant and anti-inflammatory activities and total polyphenol and flavonoid contents of Anadenanthera colubrina from northern Peru
Source: Rev Peru Med Exp Salud Publica. 2026 Mar 27;43(1):51–60. doi: 10.17843/rpmesp.2026.431.15207 (PMC13245994; doi:10.17843/rpmesp.2026.431.15207)
Supplement: Supplementary material. — Available in the electronic version of the RPMESP. [file rpmesp-43-01-15207-s001.docx]

**MATERIAL SUPLEMENTARIO**

**Figura 1.** Certificado de identificación de muestras de *Anadenanthera colubrina* emitido por el Herbarium Truxillense (HUT) de la Universidad Nacional de Trujillo (UNT)


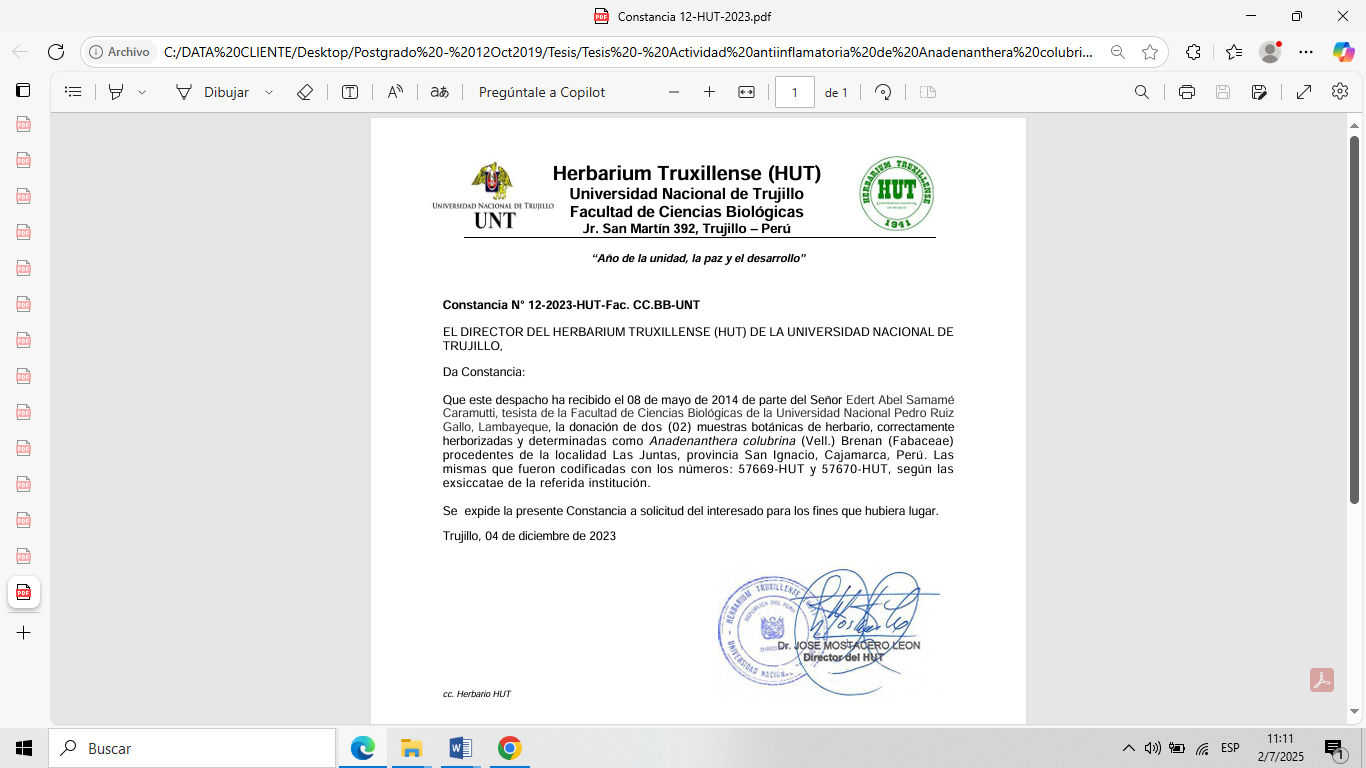


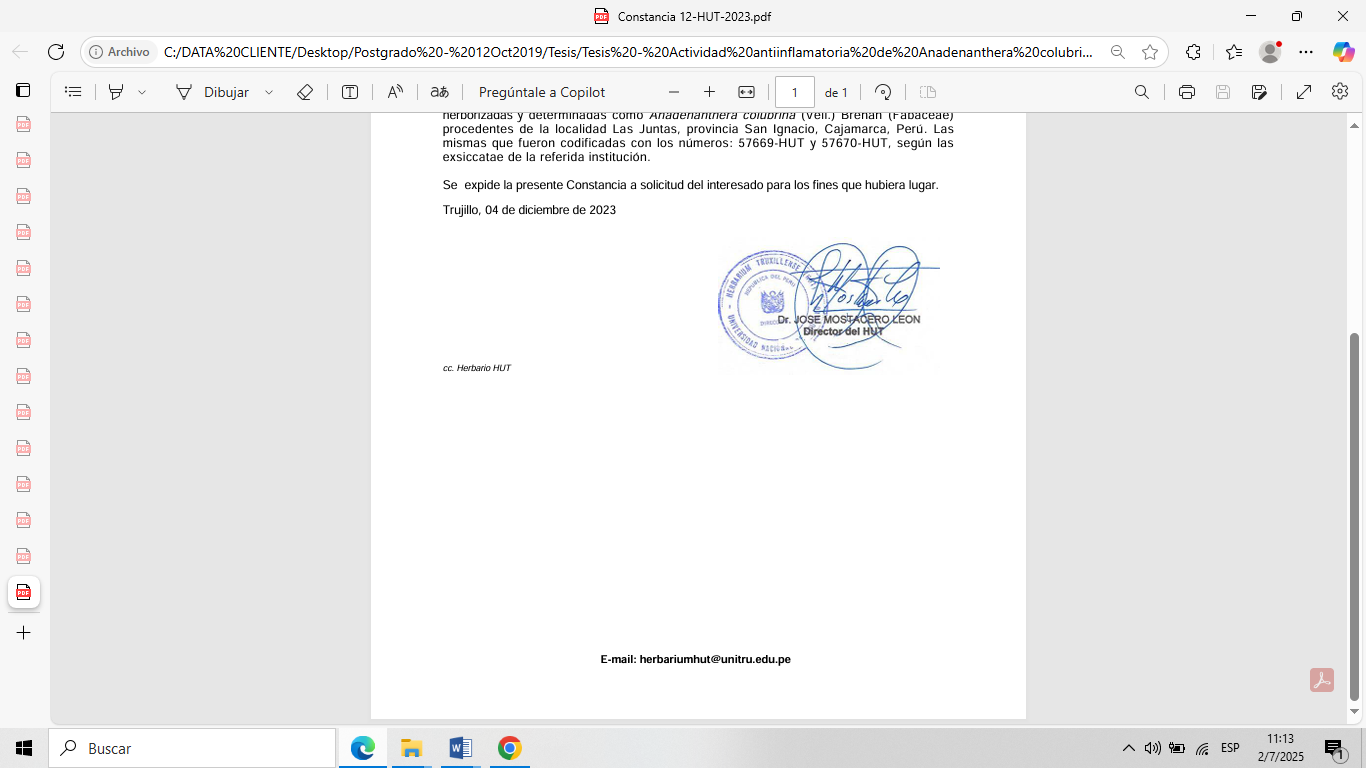


**Tabla 1.** Tamizaje fitoquímico cualitativo del extracto hidroetanólico de la corteza de *Anadenanthera colubrina* (EEAC)

| **Metabolito secundario** | **Reactivo** | **Resultado** |
| --- | --- | --- |
| Flavonoides | Shinoda | (+++) |
| Polifenoles | FeCl_3_ | (+++) |
| Taninos | Gelatina | (+++) |
| Alcaloides | Dragendorff | (-) |
|  | Mayer | (-) |
|  | Bertrand | (-) |
| Glucósidos | Vainillina – ácido sulfúrico | (+) |
| Antraquinonas y antronas | Bornträger | (+) |
| Triterpenos y/o esteroides | Liebermann – Burchard | (-) |
| Saponinas | Índice afrosimétrico | (-) |

Clave: (+): Presente; (++): Rico; (+++): Muy rico; (-): Indetectable o ausente

**Tabla 2.** Actividad captadora de radicales libres DPPH^●^ y poder antioxidante redactor férrico (FRAP) del extracto hidroetanólico de la corteza de *Anadenanthera colubrina* (EEAC) y estándares. Los resultados son presentados como media + desviación estándar (*n* = 3), representativos de dos réplicas.

| **Radical libre** | **Muestra** | **Ecuación de la recta** | **IC_50_ (μg/mL)** | **Capacidad antioxidante equivalente** |
| --- | --- | --- | --- | --- |
| DPPH^●^ | EEAC | y = 11.201x + 2.2232  *R*^2^ = 0.9959 | 4.37 + 0.13 | 747.55 + 29.6 mg equivalentes Trolox/g EEAC seco |
|  | Trolox | y = 15.523x - 1.2702  *R*^2^ = 0.999 | 3.26 + 0.05 |  |
| FRAP | EEAC | y = 0.0537x + 0.0127  *R*^2^ = 0.9936 | ^-^ | 435.88 + 13.3 mg equivalentes FeSO_4_/g EEAC seco |
|  | FeSO_4_ | y = 0.141x - 0.0103  *R*^2^ = 0.9986 | ^-^ |  |
