# Supplementary material for: In vitro antioxidant and anti-inflammatory activities and total polyphenol and flavonoid contents of Anadenanthera colubrina from northern Peru
Source: Rev Peru Med Exp Salud Publica. 2026 Mar 27;43(1):51–60. doi: 10.17843/rpmesp.2026.431.15207 (PMC13245994; doi:10.17843/rpmesp.2026.431.15207)
Supplement: Translation

# Translation: Actividades antioxidante y antiinflamatoria *in vitro* y contenidos de polifenoles y flavonoides totales de *Anadenanthera colubrina* del norte peruano

**Authors:** Edert Abel Samame-Caramutti, Luis Salcedo-Valdez, Silvia Suárez-Cunza

**Affiliations:** 1 Universidad Nacional Mayor de San Marcos, Facultad de Medicina, Instituto de Investigación de Bioquímica y Nutrición.

**Keywords:** Antinflamatorios, Antioxidantes, Flavonoides, Ensayos *in vitro*, Polifenoles

## Abstract

**Objetivos.:**

Evaluar la actividad antioxidante y antiinflamatoria *in vitro* del extracto hidroetanólico de la corteza de *Anadenanthera colubrina* del norte peruano, así como sus contenidos de polifenoles (CPT) y flavonoides (CFT) totales.

**Materiales y métodos.:**

La actividad antioxidante fue evaluada usando los ensayos químicos de captación de radicales 2,2-difenil-1-picril-hidrazilo (DPPH^●^) y de poder antioxidante reductor férrico (FRAP), así como midiendo la peroxidación lipídica (formación de sustancias reactivas al ácido tiobarbitúrico, TBARS) y hemólisis inducida por H_2_O_2_. La actividad antiinflamatoria fue determinada mediante los ensayos de desnaturalización de ovoalbúmina inducida por calor y hemólisis inducida por hipotonicidad.

**Resultados.:**

El extracto mostró una alta actividad antioxidante tanto en el ensayo de captación de radicales DPPH^●^ (747,6 mg equivalentes Trolox/gramo de extracto seco) como en el ensayo de FRAP (435,9 mg equivalentes FeSO_4_/gramo de extracto seco). El extracto (50 a 1000 μg/mL) inhibió la peroxidación lipídica, la hemólisis inducida por H_2_O_2_, y la desnaturalización de ovoalbúmina. No se observó actividad en el ensayo de hemólisis inducido por hipotonicidad. El CPT y el CFT fueron de 642,7 mg equivalentes de ácido gálico/gramo y 416,2 mg equivalentes de catequina/gramo de extracto seco, respectivamente.

**Conclusiones.:**

Estas actividades fueron reportadas por primera vez para *A. colubrina* del norte peruano, y sugiere que el extracto hidroetanólico de la corteza de *A. colubrina* posee propiedades antioxidante y antiinflamatoria preliminares significativas *in vitro* y que los altos contenidos de polifenoles y flavonoides pueden ser responsables, al menos parcialmente, de estas actividades.

## INTRODUCCIÓN

*Anadenanthera colubrina* (Vell.) Brenan es una Fabaceae sudamericana ampliamente usada en la medicina popular debido a sus conocidas propiedades antiinflamatorias ^1^ por lo que se le considera una fuente potencial de compuestos con actividad biológica.

Una de las partes más utilizadas es la corteza, principalmente en forma de jarabe, decocción, infusión y maceración. Estas preparaciones se utilizan para tratar una variedad de afecciones, que van desde problemas comunes como alergias e inflamación hasta enfermedades graves como anemia, enfermedades respiratorias (por ejemplo, bronquitis y tuberculosis) y cáncer; también sirven como cicatrizantes ^2^. En este sentido, varios estudios han reportado las propiedades antioxidantes de la corteza ^3^^,^^4^, las que pueden explicar su actividad antiinflamatoria *in vivo*^5^ e *in vitro*^6^.

*A. colubrina* crece en los bosques tropicales lluviosos y estacionalmente secos del Perú entre los 600 y 2000 m sobre el nivel del mar ^7^. Aunque esta especie ha sido ampliamente utilizada en la medicina popular sudamericana ^8^, no ha recibido la debida importancia en el norte del Perú, donde se emplea principalmente para la producción de madera ^7^. Considerando que existe escasa información científica que avale su uso tradicional ^9^ y que la variedad nativa del Perú no ha sido estudiada previamente, el objetivo de este estudio fue determinar las actividades antioxidante y antiinflamatoria *in vitro* de la corteza de *A. colubrina* del norte peruano, así como su contenido total de polifenoles y flavonoides.

**MENSAJE CLAVE**

Motivación para realizar el estudio. A pesar de su amplio uso en la medicina popular sudamericana, *Anadenanthera colubrina* del norte peruano es empleada principalmente como maderable, ignorando ampliamente su potencial medicinal.

Principales hallazgos. El extracto hidroetanólico de la corteza de *A. colubrina* presentó actividades antioxidante y antiinflamatoria preliminares sustanciales *in vitro*, atribuibles, al menos parcialmente, a sus altos contenidos de polifenoles y flavonoides.

Implicancias para la salud pública. Estos hallazgos demuestran que *A. colubrina* del norte peruano tiene potencial significativo para prevenir y tratar condiciones inflamatorias, y beneficiarán a los habitantes locales, quienes a menudo desconocen las propiedades medicinales de nuestros recursos naturales, reforzando así su valor tanto en el Perú como en el extranjero.

## MATERIALES Y MÉTODOS

### Reactivos y estándares

2,2-difenil-1-picril-hidrazilo (DPPH^●^), reactivo de fenol de Folin-Ciocalteu (2N), ácido gálico, (+)-catequina, ácido (+)-6-hidroxi-2,5,7,8-tetrametilcromano-2-carboxílico (Trolox), ácido 2-tiobarbitúrico y fosfato de sodio monobásico (> 99%) se obtuvieron en Sigma-Aldrich (MO, EEUU). Carbonato de sodio, cloruro de sodio (> 99,5%), D(+)-glucosa, Perhydrol® 30% H_2_O_2_ y fosfato disódico anhidro (99%) se compraron en Merck (Alemania). Ácido cítrico-1-hidrato se obtuvo de Riedel-de Ha*ë*n (Alemania). Diclofenaco sódico fue una generosa donación de un laboratorio farmacéutico nacional. Todos los demás productos químicos utilizados fueron de grado analítico.

Todos los valores de absorbancia fueron determinados usando un espectrofotómetro Thermo Fisher Scientific Genesys 50S UV-Vis (Waltham, MA, EEUU) en cubetas de cuarzo de 1 cm.

### Material vegetal y extracción

La corteza de *Anadenanthera colubrina* (3 kg) fue recolectada localmente en el bosque primario de Las Juntas (departamento de Cajamarca, Perú) (5°20’30” S, 78°46’00” O; altitud media 625 m) ^10^, con la autorización de los habitantes locales. La planta fue autenticada por el Prof. Eric Rodríguez-Rodríguez, y dos especímenes de referencia fueron depositados en el Herbarium Truxillense de la Universidad Nacional de Trujillo (Trujillo, Perú) (número de código de los especímenes: 57669-HUT y 57670-HUT) (material suplementario: figura 1).

El extracto hidroetanólico se obtuvo mediante métodos reportados en la literatura para otras Fabaceae ^11^^,^^12^, con modificaciones; brevemente, la corteza se secó durante 14 días a temperatura ambiente y se molió. Una muestra de corteza en polvo (1,4 kg) se extrajo mediante extracción exhaustiva sucesiva con etanol al 96% (1:2 p/v) durante 48 horas a temperatura ambiente en agitación. Los extractos crudos se combinaron, filtraron y concentraron hasta la sequedad, obteniéndose un producto seco de color marrón rojizo (extracto hidroetanólico de la corteza de *A. colubrina*, EEAC). El rendimiento de la extracción fue de 20,7%. El EEAC se almacenó a 4°C.

El EEAC se disolvió por completo en una mezcla de etanol al 96%: agua bidestilada (1:29), y esta solución se utilizó para todos los ensayos.

### Análisis fisicoquímico

El contenido de sólidos solubles del EEAC (2 mg/mL) se determinó gravimétricamente usando una balanza analítica Ohaus® Pioneer^TM^ (120 g, d = 1 mg; Parsipanny, NJ, EEUU), y midiendo el índice de refracción (disolvente: etanol al 96%: agua bidestilada, 1:29) utilizando un refractómetro de mano ATAGO® PAL-α (Tokio, Japón). El pH se obtuvo utilizando tiras indicadoras de pH (Merck, Alemania).

### Análisis fitoquímico cualitativo y contenido de polifenoles (CPT) y flavonoides (CFT) totales:

El análisis fitoquímico cualitativo se realizó según Lock de Ugaz ^13^. El CPT se determinó mediante el método de Folin-Ciocalteu ^14^. El EEAC (40-120 µg/mL, 0,1 mL) se incubó con el reactivo de Folin al 10% (0,5 mL) y Na_2_CO_3_ al 7,5% p/v (0,4 mL) a temperatura ambiente durante 30 minutos. Los cambios de absorbancia se leyeron a 765 nm. Los resultados se expresaron en mg de equivalentes de ácido gálico (EAG)/g de EEAC seco, y se calcularon a partir de una curva de calibración que utilizó ácido gálico como estándar (0-100 μg/mL) y la fórmula: 

CPT: mg EAG / g EEAC = (*A*_*s* * FD) / (*m* * *c*)

donde *A*_*s*_ es la absorbancia con la muestra (EEAC), *m* es la pendiente de la ecuación de la curva estándar de ácido gálico, *c* es la concentración de la muestra (EEAC), y FD es el factor de dilución.

El CFT se estimó como fue descrito por Zhishen *et al*. ^15^. El EEAC (50-250 µg/mL, 0,5 mL) fue incubado, sucesivamente, con NaNO_2_ al 5% (p/v; 0,15 mL) por 5 minutos, AlCl_3_ al 2,5% (p/v; 0,25 mL) durante 6 minutos y NaOH 1 M (0,25 mL) por 10 minutos. Los cambios en la absorbancia se leyeron a 510 nm. Los resultados fueron expresados como mg de equivalentes de catequina (EC)/g de EEAC seco, y se calculó a partir de una curva de calibración utilizando catequina como estándar (0-72 µg/mL) y la fórmula:

CFT: mg CE / g EEAC = (*A*_*s* * FD) / (*m* * *c*)

donde *m* es la pendiente de la ecuación de curva estándar de catequina, y *A*_*s*_ *, c,* y FD son como se indicó antes.

### Actividad antioxidante (Ensayos químicos y biológicos):

Ensayo de captación de radicales libres DPPH^●^

La actividad captadora de radicales libres DPPH^●^ fue evaluada de acuerdo con Brand-Williams *et al*. ^16^. Brevemente, una solución de DPPH^●^ (20 mg/dL) preparada en etanol fue agitada durante 40 minutos; esta solución stock fue mantenida en la oscuridad a 4°C. La absorbancia inicial de la solución de trabajo se ajustó a 0,9 + 0,02 a 517 nm (concentración de DPPH^●^: 78,5 μM) con el mismo disolvente, y fue empleada para preparar el control (absorbancia: 0,6 + 0,02). El EEAC (5-20 µg/mL, 0,4 mL) se incubó con la solución de trabajo de DPPH^●^ (0,8 mL) por 30 minutos en la oscuridad. Los cambios en la absorbancia se leyeron a 517 nm. El porcentaje de captación de radicales se calculó como:

% captación de radicales = ((*A_c* - *A_s*) * 100) / A_c

donde *A*_*c*_ y *A*_*s*_ son las absorbancias sin (control negativo) y con la muestra, respectivamente.

Los valores de concentración inhibitoria de EEAC necesarios para captar el 50% del radical libre inicial (IC_50_) se calcularon por regresión lineal. La capacidad antioxidante en equivalentes de Trolox (CAET-DPPH^●^) se expresó como mg ET/g de EEAC seco, y se calculó a partir de una curva de calibración utilizando Trolox como estándar (0-15 µg/mL) y la fórmula:

CAET-DPPH^●^: mg ET / g EEAC = IC_50 Trolox / IC_50 EEAC

Ensayo de poder antioxidante reductor férrico (FRAP)

El ensayo se realizó de acuerdo con Benzie y Strain ^17^. El EEAC (40-160 μg/mL, 50 μL) fue añadido a 950 μL de la solución de trabajo de FRAP (10 mM 2,4,6-tris(2-piridil)-s-triazina (TPTZ) en HCl 40mM, FeCl_3_ 20 mM en agua bidestilada, y tampón acetato 0,3 M, pH 3,6, en una proporción de 1:1:98). Las mezclas de reacción se incubaron durante 10 min. Los cambios en la absorbancia se leyeron a 593 nm. Los resultados fueron expresados como mg equivalentes de FeSO_4_/g de EEAC seco, y se calcularon a partir de una curva de calibración utilizando FeSO_4_ como estándar (0-91 µg/mL) y la fórmula:

FRAP: mg Eq FeSO_4 / g EEAC = (*A_s* * FD) / (*m* * c)

donde *A*_*s*_ es la absorbancia con la muestra (EEAC), *m* es la pendiente de la ecuación de la curva estándar de FeSO_4_, c es la concentración de la muestra (EEAC), y FD es el factor de dilución.

Ensayo de inhibición de la peroxidación lipídica usando sustancias reactivas al ácido tiobarbitúrico (TBARS)

El estrés oxidativo, expresado como TBARS, se midió por el método de Buege y Aust ^18^, con modificaciones. El tejido hepático congelado de una rata Holtzman (previamente perfundido con KCl 0,154 M helado) fue homogeneizado en solución salina tamponada con fosfato fría (PBS, pH 7,4). La peroxidación lipídica se inició añadiendo al homogenizado de hígado al 5% (p/v, 0,9 mL), sucesivamente, ácido ascórbico 4 mM (0,045 mL) y FeSO_4_ 2 mM (0,015 mL). Posteriormente, se incluyeron diferentes concentraciones de EEAC (100-1000 μg/mL, 0,06 mL) o PBS (pH 7,4, 0,06 mL y 0,12 mL para los grupos de estrés inducido y control, respectivamente) en las mezclas de reacción; luego, estas fueron incubadas por 30 minutos a temperatura ambiente. Después, cada mezcla de reacción (0,3 mL) se mezcló con ácido tricloroacético al 20% (0,6 mL), se calentó en agua hirviendo durante 15 minutos, se enfrió con agua corriente, se mezcló con ácido tiobarbitúrico al 0,67% (TBA en HCl 0,25 N, 0,9 mL) y se calentó de nuevo en agua hirviendo durante 30 minutos. Tras enfriar con agua corriente fría, cada tubo se centrifugó (5488 x g durante 10 minutos), se recogió el sobrenadante y se determinó la absorbancia a 535 nm.

La actividad antioxidante se expresó como porcentaje de inhibición de la peroxidación lipídica y como concentración de TBARS. El porcentaje de inhibición de la peroxidación lipídica se calculó de la siguiente manera:

% inhibición de peroxidación lipídica = ((*A_c* - *A_s*) * 100) / A_c

donde *A*_*c*_ es la absorbancia del grupo de estrés inducido (0% de inhibición de peroxidación lipídica, *control negativo*) y *A*_*s*_ es la absorbancia con la muestra (EEAC).

La concentración de TBARS se expresó como nmol de TBARS/mg de tejido hepático y se calculó de la siguiente manera:

TBARS: (nmol TBARS/mg tejido hepático) = (*A_s* * V_t ) /

(*ε* * l * V_s * C_H)

donde *A*_*s*_ es la absorbancia de cada mezcla de reacción, Vt es el volumen final de la mezcla de reacción, *ε* es el coeficiente de absorción molar del complejo MDA-TBA_2_ a 535 nm (1,56 x 10^5^ M^-1^ cm^-1^), l es el paso óptico de la cubeta (1 cm), V_s_ es el volumen de muestra en la mezcla de reacción, CH es la concentración de homogenizado de hígado de rata.

Ensayo de hemólisis oxidativa inducida por H_2_O_2_

El ensayo se realizó siguiendo el método de Xu *et al*. ^19^, con modificaciones. Se empleó sangre venosa humana fresca (3 mL) de un donante sano, y se diluyó con solución esterilizada de Alsever (dextrosa al 2%, citrato de sodio al 0,8%, ácido cítrico al 0,05% y NaCl al 0,42% en agua bidestilada). La solución fue centrifugada (625 x g por 3 minutos) y lavada tres veces con el mismo volumen de vehículo (PBS frío 1X, pH 7,4) para separar los glóbulos rojos humanos (GR) de la capa de glóbulos blancos, el plasma y la hemoglobina libre (Hb) liberada de los GR lesionados. Una suspensión de GR al 5% (en solución de Alsever’s helada, 0,1 mL) fue mezclada con EEAC (50-150 μg/mL, 0,45 mL) o vehículo (0,45 mL y 0,9 mL para los grupos de estrés inducido y de control, respectivamente). Las mezclas de reacción se preincubaron a 37 °C durante 15 minutos. Luego, se añadió H_2_O_2_ 166 mM (en vehículo, 0,45 mL), la mezcla de reacción se incubó a 37°C durante 4 horas y se centrifugó a 625 x g durante 10 minutos. El contenido de Hb en el sobrenadante se estimó en 405 nm. Después de cada ensayo, la solución de GR se almacenó a 4°C durante un máximo de 72 h para garantizar la viabilidad y el nivel de antioxidantes celulares. El porcentaje de prevención de hemólisis se calculó de la siguiente manera:

% prevención de hemólisis = ((*A_c* - *A_s*) * 100) / *A_c*

donde *A*_*c*_ es la absorbancia del grupo de estrés inducido (0% de prevención de hemólisis, control negativo) y *A*_*s*_ es la absorbancia con la muestra (EEAC).

### Actividad antiinflamatoria

Inhibición de la desnaturalización de ovoalbúmina

El ensayo se llevó a cabo según lo descrito por Mizushima y Kobayashi ^20^ y Chandra *et al*. ^21^, con modificaciones. La mezcla de reacción (1,3 mL) consistió en ovoalbúmina fresca: PBS (pH 6,4) (1:1, 0,1 mL), PBS (pH 6,4, 0,7 mL) y diferentes concentraciones del EEAC (125-750 μg/mL, 0,5 mL). Se usaron agua bidestilada y diclofenaco sódico (0,5-3 mg/mL) para el grupo de estrés inducido y como estándar, respectivamente. Las mezclas de reacción se incubaron a 37 + 1°C durante 20 minutos y posteriormente fueron calentadas a 65 + 2°C por 5 minutos para inducir la desnaturalización. Después de enfriar, los cambios en la absorbancia se leyeron a 660 nm. El porcentaje de inhibición de la desnaturalización de proteínas se calculó como:

% inhibición de desnaturalización de proteínas = ((*A_c* - *A_s*) * 100) / *A_c*

donde *A*_*c*_ es la absorbancia del grupo de estrés inducido (0% de inhibición de desnaturalización de proteínas, control negativo) y *A*_*s*_ es la absorbancia con la muestra.

Estabilización de membrana de glóbulos rojos humanos (GR)

El ensayo se realizó de acuerdo con los procedimientos descritos por Lavanya *et al*. ^22^ y Torres Carro *et al*. ^23^, con modificaciones. Se empleó sangre venosa humana fresca (3 mL) de un donante sano, y se diluyó con un volumen igual de solución esterilizada de Alsever. La solución fue centrifugada (5,488xg por 10 minutos) y el paquete celular fue lavado cinco veces con un volumen igual de solución salina isotónica (NaCl al 0,85%, pH 7,2) para preparar una suspensión de GR con solución salina isotónica al 10% (v/v). La suspensión fue almacenada a 4°C. La mezcla de reacción (1,5 mL) consistió de la suspensión de GR al 10% (0,2 mL), tampón fosfato sódico 0,14 M (pH 7,4, 0,333 mL), y concentraciones diferentes del EEAC (250-750 μg/mL, 0,267 mL). Se empleó agua bidestilada (0,7 mL) para inducir la hemólisis hipotónica. Se usaron vehículo (etanol al 96%: agua bidestilada, 1:29) y diclofenaco sódico (1-3 mg/mL) para el grupo de estrés inducido y como estándar, respectivamente. Las mezclas de reacción fueron incubadas a 37 + 1°C por 30 minutos y luego centrifugadas a 650 x g por 3 minutos. El contenido de Hb en el sobrenadante se midió a 550 nm. Después de cada prueba, la solución GR se almacenó a 4°C durante un máximo de 72 h para garantizar la viabilidad celular. El porcentaje de prevención de hemólisis se calculó de la siguiente manera:

% prevención de hemólisis = ((*A_c* - *A_s*) * 100) / *A_c*

donde *A*_*c*_ es la absorbancia del grupo de estrés inducido (0% prevención de hemólisis, control negativo) and *A*_*s*_ es la absorbancia con la muestra.

Análisis estadísticos

Los ensayos se realizaron en tres experimentos independientes, cada uno realizado por duplicado o triplicado. Todos los análisis se realizaron usando Minitab Statistical Software y GraphPad Prism (versión 8.0.1). La normalidad y homogeneidad de las varianzas de los datos se evaluó mediante las pruebas de Shapiro-Wilk y de Levene, respectivamente (p>0,05 en ambas). Dado que los datos estaban distribuidos normalmente, se utilizó un ANOVA de un factor seguido de la prueba post-hoc de Tukey. Los resultados se expresan como media + desviación estándar, y la significancia estadística se definió como p<0,05.

Aspectos éticos

El estudio se llevó a cabo de acuerdo con la Declaración de Helsinki ^24^ y las normas éticas establecidas. Se obtuvo una muestra de sangre humana de un voluntario sano en el Instituto de Investigación de Bioquímica y Nutrición (IIBN), UNMSM, después de la recepción de un consentimiento informado por escrito. Con respecto al componente animal, utilizamos tejido hepático de una rata sin tratamiento previo como uso secundario de muestras de un proyecto paralelo del IIBN, siguiendo los principios de las 3Rs (Reemplazo, Reducción y Refinamiento) para evitar el sacrificio de animales adicionales. Este proyecto fue aprobado por el Comité de Ética de la Facultad de Medicina de la UNMSM (Aprobación de ética No. 0185-2024), de acuerdo con el Acta de Evaluación Ética de Estudios de Investigación.

## RESULTADOS

### Análisis fisicoquímico

El contenido de sólidos solubles fue de 1,606 + 0,3 mg/mL y de 1,711 + 0,2 mg/mL, según el índice de refracción y el análisis gravimétrico, respectivamente. El pH fue de 5,5-6,0 (ligeramente ácido).

### Análisis fitoquímico cualitativo, CPT y CFT

Como se presenta en el material suplementario: tabla 1, el EEAC tiene un alto contenido de compuestos fenólicos (taninos y flavonoides), bajo contenido de glucósidos, antraquinonas y antronas, y presencia indetectable o nula de alcaloides, triterpenos, esteroides y saponinas.

La determinación de polifenoles y flavonoides totales confirmó su abundancia, con 642,7 + 17 mg (3,78 + 0,1 mmol) EAG/g y 416,2 + 20 mg (1,6 + 0,08 mmol) EC/g extracto seco, respectivamente; esto es, el CPT representa aproximadamente el 65% en EAG del extracto, que incluye principalmente flavonoides (alrededor del 41% en EC del extracto). La relación flavonoides/polifenoles fue de 0,65.

### Actividad antioxidante (ensayos químicos y biológicos)

Ensayo de captación de radicales libres DPPH^●^

El EEAC mostró un valor de IC_50_ (4,37 µg/mL) cercano al estándar Trolox (3,26 µg/mL, figura 1 y material suplementario: tabla 2). El valor mencionado anteriormente representa una CAET-DPPH^●^ de 747,55 mg (2,99 mmol) equivalentes de Trolox/g EEAC seco (material suplementario: tabla 2).

**Figura 1:** Efectos de captación de los radicales libres DPPH^●^ (78,5 μM) del extracto hidroetanólico de la corteza de *Anadenanthera colubrina* (EEAC) y Trolox. Los resultados son presentados como media + desviación estándar (n = 3), representativos de dos réplicas.

Ensayo de FRAP

El EEAC exhibió una capacidad antioxidante de 435,88 mg (2,87 mmol) equivalentes de FeSO_4_/g EEAC seco (figura 2 y material suplementario: tabla 2).

**Figura 2:** Reactividad del extracto hidroetanólico de la corteza de *Anadenanthera colubrina* (EEAC) y sulfato ferroso en el ensayo de poder antioxidante redactor férrico (FRAP). Los resultados son presentados como media + desviación estándar (n = 3), representativos de dos réplicas.

Ensayo de inhibición de la peroxidación lipídica usando TBARS

Todas las concentraciones probadas (100, 500, y 1000 μg/mL) fueron eficaces para reducir la peroxidación lipídica de manera dependiente de la dosis, en un 22, 59 y 68%, respectivamente, con respecto al grupo de estrés inducido (EI) (figura 3a). No se encontraron diferencias entre el tratamiento con el EEAC a 1000 μg/mL y el grupo Control (72%).

Cuando se expresó como nmol/mg de tejido hepático, la producción de TBARS fue mayor en el grupo EI que en los tratamientos con EEAC a 500 y 1000 μg/mL, indicando una dependencia de la concentración del extracto. No se encontraron diferencias entre el grupo EI y el EEAC a 100 μg/mL, ni entre el Control y los tratamientos con EEAC a 500 y 1000 μg/mL (figura 3b).

**Figura 3:** (a) Porcentaje de inhibición de la peroxidación lipídica por el extracto hidroetanólico de la corteza de *Anadenanthera colubrina* (EEAC) y (b) efecto del EEAC en la formación de TBARS en homogeneizado de hígado de rata. Los resultados son presentados como media + desviación estándar (n = 3), representativos de dos réplicas, y se evaluaron mediante análisis de varianza de un factor y la prueba de Tukey (p<0,05). Las diferencias estadísticas se indican con letras diferentes. EI: estrés inducido.

Ensayo de hemólisis oxidativa inducida por H_2_O_2_

El EEAC, administrado a 50, 100, and 150 μg/mL, disminuyó la hemólisis causada por H_2_O_2_ cuando se comparó con el grupo EI (figura 4). La prevención de la hemólisis dependió de la dosis únicamente para los tratamientos con 50 y 100 μg/mL (inhibición del 49 y 75%, respectivamente) dado que el tratamiento con 150 μg/mL provocó una inhibición (65%) similar a la observada para el grupo de 100 μg/mL. De modo interesante, el porcentaje de inhibición para las tres concentraciones de EEAC fue mucho mayor que el reportado para el grupo Control (18%).

**Figura 4:** Inhibición de la hemólisis oxidativa por H_2_O_2_ por el extracto hidroetanólico de la corteza de *Anadenanthera colubrina* (EEAC). Los resultados son presentados como media + desviación estándar (n = 3), representativos de tres réplicas, y se evaluaron mediante análisis de varianza de un factor y la prueba de Tukey (p<0,05). Las diferencias estadísticas se indican con letras diferentes. EI: estrés inducido.

### Actividad antiinflamatoria

Inhibición de la desnaturalización de ovoalbúmina

Se observó una inhibición dependiente de la dosis de la desnaturalización de ovoalbúmina con el EEAC a 125, 250 y 500 μg/mL, en 34, 42, y 47%, respectivamente, con respecto al grupo EI (figura 5). Curiosamente, el extracto a 750 μg/mL redujo la desnaturalización solo en un 40%, menor que la inhibición observada a 500 μg/mL. En cualquier caso, el EEAC en todas las concentraciones analizadas mostró una inhibición mayor que el fármaco antiinflamatorio no esteroideo (AINE) diclofenaco sódico a 0,5 y 1 mg/mL (19 y 22%, respectivamente), pero menor que dicha referencia a 3 mg/mL (58%). El EEAC a 250 y 500 μg/mL mostró una mejor inhibición de la desnaturalización de proteínas que el diclofenaco sódico a 2 mg/mL.

**Figura 5:** Inhibición de la desnaturalización de ovoalbúmina por el extracto hidroetanólico de la corteza de *Anadenanthera colubrina* (EEAC). Diclofenaco sódico (DS) fue usado como estándar. Los resultados son presentados como media + desviación estándar (n = 3), representativos de tres réplicas, y se evaluaron mediante análisis de varianza de un factor y la prueba de Tukey (p<0,05). Las diferencias estadísticas se indican con letras diferentes. EI: estrés inducido.

Estabilización de membrana de GR

El EEAC a 250, 500, y 750 μg/mL no tuvo un efecto significativo sobre la hemólisis inducida por hipotonicidad. En este aspecto, el extracto a 250 y 500 μg/mL produjo una hemólisis ligeramente mayor que la del grupo EI (6 y 5%, respectivamente). Asimismo, la concentración de 750 μg/mL inhibió la hemólisis solo en un 6%. A pesar de ello, ninguna de las concentraciones mostró una diferencia significativa con respecto al grupo EI. Por otro lado, el diclofenaco sódico inhibió la hemólisis entre un 29% y un 42% a las concentraciones utilizadas (1, 2, y 3 mg/mL).

## DISCUSIÓN

Los resultados acerca del contenido fitoquímico del EEAC son consistentes con informes previos sobre la corteza de *A. colubrina*, es decir, el extracto hidroalcohólico es rico en compuestos fenólicos ^4^^,^^25^, que incluyen tanto taninos como flavonoides ^6^. Sin embargo, algunos estudios previos son inconsistentes con los resultados de este análisis fitoquímico. Silva *et al*. ^26^ reportaron la presencia de alcaloides y saponinas, así como la ausencia de flavonoides, antraquinonas y glucósidos en el extracto hidroalcohólico de la corteza. Por otro lado, Sá *et al*. ^27^ revelaron que el extracto hidroalcohólico de la corteza contiene compuestos fenólicos, taninos y azúcares, pero no se detectaron flavonoides, esteroides ni terpenoides. Adicionalmente, Pessoa *et al*. ^28^ reportaron la presencia de azúcares reductores, saponinas, triterpenos y esteroides. Estas diferencias indicarían que las condiciones edáficas y ambientales, así como las diferencias en los procedimientos de extracción, afectan la biosíntesis o la presencia de metabolitos secundarios a pesar de tratarse de la misma especie.

Curiosamente, el extracto hidroetanólico de la corteza de la variedad brasileña reportada por Mota *et al*. ^4^ mostró valores de CPT y CFT más altos que aquellos de la variedad de *A. colubrina* nativa del norte peruano investigada en este estudio (682 mg EAG/g extracto y 445 mg EC/g extracto para la variedad brasileña vs. 643 mg EAG/g extracto y 416 mg EC/g extracto para la variedad peruana, respectivamente). A pesar de ello, la variedad peruana tiene mayores propiedades antioxidantes que la variedad brasileña antes mencionada.

El EEAC mostró un buen comportamiento en dos ensayos antioxidantes con diferentes mecanismos de acción, la transferencia de un solo electrón para el ensayo FRAP y la transferencia de un átomo de hidrógeno o de un solo electrón para el ensayo de captación de radicales DPPH^● (^^29^. Es más, como reportaron Desmarchelier *et al*. ^30^, el EEAC disminuyó la formación de malondialdehído y otros TBARS como subproductos de la peroxidación lipídica en homogeneizados de hígado de rata y, además, redujo el daño oxidativo causado por H_2_O_2_ en un modelo de GR, mostrando la capacidad del EEAC de disminuir el estrés oxidativo *in vitro*.

Estos resultados sugieren que la variedad peruana de *A. colubrina* posee la actividad antioxidante previamente reportada para la corteza. Esta actividad antioxidante probablemente se deba al contenido fitoquímico del EEAC, principalmente compuestos fenólicos (taninos y flavonoides) ^4^. No obstante, existen algunos resultados contradictorios respecto a la producción de polifenoles por *A. colubrina* en diferentes condiciones ambientales ^31^, lo que a su vez podría explicar algunas discrepancias entre el presente estudio y reportes previos ^4^^,^^26^^,^^32^.

Es de destacar que la variedad peruana exhibió un valor de CAET-DPPH^●^ más alto y un IC_50_ más bajo que la variedad brasileña reportada por el estudio antes mencionado de Mota *et al*. ^4^ (269 mg equivalentes de Trolox/g extracto y 13 μg/mL para la variedad brasileña vs. 748 mg equivalentes de Trolox/g extracto y 4 μg/mL para la variedad peruana, respectivamente). En consecuencia, las propiedades antioxidantes (CAET-DPPH^●^ y IC_50_) de la variedad brasileña se considerarían sólo moderadamente intensas ^4^, e inferiores a las de la variedad peruana reportada aquí.

Estos hallazgos indican que, como se mencionó previamente, además de las diferencias en las técnicas de extracción, las condiciones edáficas y ambientales influyen en la presencia de metabolitos secundarios y, en consecuencia, en la actividad biológica de los extractos. Esto es particularmente evidente al comparar estos resultados con los estudios de Mota *et al*. (2017) ^4^ y Silva *et al*. (2020) ^26^ mencionados anteriormente, ya que todos estos estudios utilizaron extractos hidroetanólicos. Cabe destacar el informe de Silva *et al*. (2020) ^26^, el cual reveló la presencia de compuestos ausentes en el EEAC, así como la ausencia de flavonoides, que fueron abundantes aquí. Además, su extracto exhibió un IC_50_ de 20 μg/mL en el ensayo de DPPH, mucho más alto que los 4,37 μg/mL reportados en el presente trabajo.

Los diferentes métodos de extracción también contribuyen a las variaciones en el contenido fitoquímico y la actividad antioxidante. En este contexto, varios estudios han reportado la actividad de los extractos hidrometanólicos de la corteza de *A. colubrina*; por ejemplo, Sá *et al*. (2016) reportaron una extracción reducida de flavonoides ^27^, los cuales fueron abundantes en el presente estudio. Adicionalmente, Pessoa *et al*. (2012) detectaron moléculas como saponinas, triterpenos y esteroides ^28^ que estuvieron ausentes en esta investigación. Este tipo de extracto también mostró una mayor actividad antioxidante en el ensayo de TBARS (IC_50_ de 62 μg/mL) ^30^ comparado con el presente estudio (IC_50_ > 100 μg/mL), pero menor actividad en el ensayo de DPPH (IC_50_ de 73 μg/mL) ^32^.

El uso de extractos acuosos de la corteza de *A. colubrina* también arrojó resultados interesantes. En concreto, Damascena *et al*. (2014) ^3^ revelaron un IC_50_ de 8,63 μg/mL en el ensayo de DPPH, indicando una menor actividad antioxidante en comparación con este trabajo. Por el contrario, los extractos acuosos mostraron actividades antioxidantes variables en el ensayo de TBARS, las cuales fueron similares ^3^ o mayores ^30^ que las observadas con el EEAC.

Se empleó el ensayo de desnaturalización de ovoalbúmina para evaluar la actividad antiinflamatoria de *A. colubrina*. Este método se basa en la acción estabilizadora de los AINE sobre la coagulación de proteínas inducida por calor ^20^. Dado que la desnaturalización de las proteínas tisulares es capaz de iniciar respuestas inmunes y causar trastornos inflamatorios, se ha sugerido que los compuestos que previenen la desnaturalización de las proteínas podrían tener potencial antiinflamatorio ^33^. En este aspecto, el EEAC previno la desnaturalización de la ovoalbúmina inducida por calor en mayor grado que el AINE diclofenaco sódico, que tiene una actividad protectora conocida contra la desnaturalización proteica.

Para corroborar este efecto antiinflamatorio, se realizó el ensayo de estabilización de la membrana de GR. Este ensayo se basa en la similitud entre las membranas de los eritrocitos y los lisosomas. La estabilización de la membrana lisosomal es deseable, ya que los lisosomas pueden liberar sus enzimas hidrolíticas en los tejidos afectados durante la respuesta inflamatoria. El efecto de cualquier extracto sobre la estabilización de los eritrocitos podría extrapolarse a la estabilización de la membrana lisosomal y, por lo tanto, la actividad antiinflamatoria de un extracto puede evaluarse mediante la prevención de la hemólisis ^34^.

No se observó actividad a las mismas concentraciones empleadas en el ensayo de desnaturalización; por el contrario, el EEAC aumentó la hemólisis inducida por hipotonicidad, aunque estos resultados no fueron del todo inesperados. El EEAC es rico en taninos (material suplementario: tabla 1), que podrían tener actividad hemolítica sobre los GR ^35^. En este sentido, Rocha *et al*. ^25^ reportaron que el extracto hidroalcohólico de la corteza de *A. colubrina* fue ligeramente tóxico para los GR, incluso a bajas concentraciones (0,25 a 32 mg/mL). Desde esta perspectiva, el alto contenido de taninos del EEAC podría explicar la hemólisis observada en el presente estudio. Tal como describieron Deng *et al*. (2019) ^35^, las altas concentraciones de algunos taninos podrían provocar cambios en la morfología de los eritrocitos y hemólisis, posiblemente debido a los grupos hidroxilo fenólicos de los taninos, que se adsorben en la membrana celular, causando deformaciones que conducen a su ruptura. Es bien sabido que los taninos se unen a las proteínas y los lípidos de la membrana celular y los alteran, modificando así su permeabilidad y provocando daño celular y lisis, lo que también afecta la coagulación sanguínea.

Aunque estos resultados indicarían que el EEAC es una fuente potencial de compuestos antiinflamatorios, especialmente aquellos relacionados con la desnaturalización de proteínas, se debe considerar que los ensayos se realizaron *in vitro*, y no necesariamente muestran lo que ocurriría *in vivo*, ya que debe considerarse la biodisponibilidad de compuestos activos, como los taninos. Sin embargo, es prometedor que *A. colubrina* haya demostrado una mejor actividad inhibidora que un AINE como el diclofenaco sódico, que puede inhibir la desnaturalización de proteínas de una manera dependiente de la dosis.

En conclusión, se reportaron por primera vez las propiedades biológicas de *Anadenanthera colubrina* del norte del Perú. En este sentido, el extracto hidroetanólico de la corteza (EEAC) posee una fuerte actividad antioxidante preliminar *in vitro*, así como un alto contenido de polifenoles y flavonoides totales. Además, el EEAC evitó la desnaturalización de la ovoalbúmina inducida por calor en mayor medida que el AINE diclofenaco sódico. Estos resultados sugieren que *A. colubrina* del norte del Perú es una buena fuente de compuestos con actividad biológica, lo que beneficia a los habitantes de esas zonas, quienes podrían desconocer las propiedades medicinales de sus propios recursos. Además, se proporciona una metodología práctica para evaluar las actividades antioxidantes y antiinflamatorias de las plantas medicinales peruanas.

Como recomendación adicional para el uso de *A. colubrina*, se sugiere que el exceso de saponinas y taninos se eliminen mediante métodos de extracción estandarizados para optimizar sus propiedades biológicas, ya que estos compuestos, aunque están presentes en la corteza, pueden ejercer efectos tóxicos en altas concentraciones. Se necesitan más investigaciones para determinar la seguridad y los mecanismos de acción específicos de *A. colubrina*, y para purificar e identificar las posibles moléculas responsables de la actividad farmacológica observada.
